# Affinity Selection-Mass Spectrometry for the Identification of Ligands of Acetylcholinesterase from *Topsentia ophiraphidites* and Docking Studies for the Dereplicated Ligands

**DOI:** 10.1021/acsomega.5c06784

**Published:** 2025-10-14

**Authors:** Larissa Ramos Guimarães da Silva, Christyne Barros de Sá, Bruno Sergio do Amaral, Nelilma Correia Romeiro, Quezia Bezerra Cass, Alessandra Leda Valverde

**Affiliations:** † Laboratório de Produtos Naturais (LaProMar), Instituto de Química, 28110 Universidade Federal Fluminense, Niterói, RJ 24020-005, Brazil; ‡ Laboratório Integrado de Computação Científica − LICC, Centro Multidisciplinar da UFRJ Macaé, 28125 Universidade Federal do Rio de Janeiro, Macaé, RJ 27930-560, Brazil; § 119519 Instituto Federal de Educação, Ciência e Tecnologia de São Paulo, Campus Pirituba, São Paulo, SP 01109-010, Brazil; ∥ Separare, Departamento de Química, 67828 Universidade Federal de São Carlos, São Carlos, SP 13565-905, Brazil

## Abstract

Acetylcholinesterase (AChE) inhibition has been successful for the treatment of Alzheimer’s disease and still stands as an important target in the search for novel ligands. In this context, affinity selection-mass spectrometry (AS-MS) has been acknowledged as a high-throughput screening (HTS) technique for large molecular libraries in drug discovery programs and natural product investigations. In this work, an AS-MS assay with AChE immobilized onto magnetic beads (AChE-MB) has been used to search for ligands in samples of the sponge *Topsentia ophiraphidites* collected in the archipelago of Fernando de Noronha, Brazil. Ligand dereplication disclosed 6-desmethyl-6-ethyl-9,10-dihydrospongosoritin A, 3,5-dibromo-*O*-methyltyrosine, 3-bromo-5-iodo-*O*-methyltyrosine, and 3,5-di-iodo-*O*-methyltyrosine as AChE ligands, which showed affinity ratios of 1.84, 1.34, 1.26, and 1.20, respectively, in the AS-MS assay. As a complementary approach, molecular docking analysis with human AChE has been carried out for the disclosed dereplicated ligands, in which the (*R*, *R*) stereoisomer of 6-desmethyl-6-ethyl-9,10-dihydrospongosoritin A stood out, performing important intermolecular interactions with the active sites of AChE, especially with the peripheral anionic site, at the entrance of the gorge. The results stimulate further investigations of these ligands in other pharmacological assays in order to better understand their mechanisms of action.

## Graphical abstract

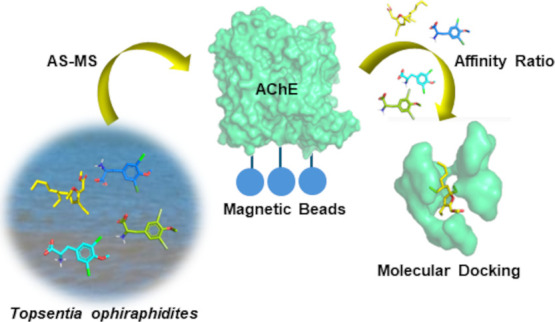

## Introduction

Natural product libraries are composed of exquisite 3D molecular structures with biological activity and have been used for prospecting ligands for a variety of molecular targets, including acetylcholinesterase (AChE). In this context, it is well-known that the inhibition of AChE has granted mitigation of Alzheimer’s disease (AD) symptoms and other neurological diseases as well.^1^ In this regard, the search for AChE ligands has been pursued mostly in terrestrial natural products, although marine libraries have shown to be an important source of AChE ligands.^2^

Affinity selection-mass spectrometry (AS-MS) has been acknowledged as a high-throughput screening (HTS) technique for large molecular libraries in drug discovery programs. In this approach, ligands can be identified by specific binding to a biological target without a label since they are disclosed by their exact mass. It is a nonfunctional assay and, as such, it has become an important tool to explore the chemical diversity of complex libraries.^3,4^ Although it has been mainly employed for synthetic libraries, it has been receiving increased attention related to natural library applications.^5,6^

The use of synthetic libraries allows better control of the chemical space, and since they are formed of known molecules, the structural characterization can be made by simple correlation of the ligand’s retention time, isotopic pattern, and exact mass. Meanwhile, the annotation of ligands identified from natural product libraries has the complexity of a nontarget analysis and thus requires, besides high-resolution mass spectrometry (HRMS), software for data processing and curation, fragmentation experiments, spectral libraries, and molecular networks. With all that, it is not always successful to chemically characterize the identified ligands from an AS-MS assay.^4,5^

AS-MS can be carried out in a variety of assay formats and with the target protein in solution or immobilized. In our research group, AChE immobilized onto magnetic beads (AChE-MB) has been efficiently used to search for ligands in natural product libraries.^7–9^

It is important to say that the affinity assay can disclose ligands that can act in a diversity of mechanisms, such as allosteric or orthosteric ligands, inhibitors, or activators, and further assays are, thus, required to elucidate their action.

In addition to experimental work, structure-based techniques like molecular docking have been thoroughly reported in the literature aiming at unraveling intermolecular interactions between ligands and AChE for proposition of binding modes and structure–activity relationships, while it may also guide further molecular modifications.^10^

The marine sponge *Topsentia ophiraphidites* occurs in the Atlantic Ocean, with records in the Caribbean and along the Brazilian coast, where it is the only taxonomically characterized representative of the genus *Topsentia*.^11,12^ The genus is well recognized for its chemical diversity, biosynthesizing metabolites from a wide range of classes, including alkaloids (with indole- or alkylpyridine-based skeletons), fatty acids, oxylipins, nitrogenous bases, terpenes, and steroids, the latter representing the most abundant class within the genus and particularly for *T. ophiraphidites*, which to date has yielded only sterols and fatty acids. Regarding the biological activities of these compound classes, indole alkaloids, generally referred to as topsentins or spongotins, have been reported to exhibit cytotoxic, antimicrobial, antiviral, and anti-inflammatory properties.^13–15^ Oxylipins, oxygenated derivatives of fatty acids, have shown cytotoxic activity against various cancer cell lines.^16^

Despite the chemical richness of the genus, *T. ophiraphidites* remain underexplored. To date, only three bioactivity studies involving specimens collected along the Brazilian coast have been reported, being the isolation and biological evaluation of halistanol A trisulfate,^17^ cytotoxic activity against colorectal (HCT-116) and breast (MCF-7) cancer cell lines,^18^ and antibacterial activity against *Staphylococcus aureus* (HU25), *Staphylococcus epidermidis* (ATCC 12228), and *Escherichia coli* .^19^ Importantly, extracts of *T. ophiraphidites* collected in the Caribbean (Curaçao) demonstrated significant AChE inhibitory activity when screened among 63 marine sponge extracts.^20^ Although the active metabolites were not identified in that study, the metabolic potential of the genus *Topsentia*, particularly for indole alkaloids, sesquiterpenes, and steroids (compound classes), already associated with AChE inhibition^21^ highlights *T. ophiraphidites* as a promising but still overlooked source of novel AChE ligands.

Herein, we describe the results of an AS-MS assay with AChE-MB for the search for ligands in samples of the sponge *T. ophiraphidites* collected in the archipelago of Fernando de Noronha, Brazil. Also, molecular docking analysis with human AChE has been carried out for the disclosed dereplicated ligands aiming at proposing intermolecular interactions.

## Experimental Section

### Sponge Material and Crude Extract Preparation

*Topsentia ophiraphidites* (FN 058) was collected in 1998 in Frade (15 m) at Fernando de Noronha Archipelago (PE, Brazil, 3° 51′ 37″ S, 32° 24′ 4″ W). Voucher specimen (21327) was deposited at the Porifera collection of Museu Nacional, Universidade Federal do Rio de Janeiro, Brazil (MNRJ). This project is registered by SISGEN under the code AB724BB. The remaining biological material was stored at – 20 °C until extraction. Individual species samples were extracted once with ethanol for 7 days and then twice with a mixture of ethyl acetate/methanol 1:1 (v/v) for 7 days. The solvent was removed at reduced pressure, affording the crude organic extracts.

### Evaluation of the AChE Potential of *Topsentia ophiraphidites* Extract

The evaluation of AChE inhibitory activity by the extract of *T. ophiraphidites* (FN 058) was carried out in a spectrophotometer using an adapted method based on the Ellman assay.^22^ The indirect test consists of the reaction between Ellman’s reagent [5,5′-dithiobis­(2-nitrobenzoic acid) or DTNB] and thiocholine, the enzymatic product of the hydrolysis of the substrate acetylthiocholine. The final product of the assay is 2-nitrobenzoate-5-mercaptothiocholine, detected by absorption at 412 nm.

In a 3 mL cuvette, 2483 μL of Tris-HCl buffer (50 mM, pH 8.0), 150 μL of methanolic sponge extract solution, 50 μL of AChE solution (5 U mL^–1^), and 100 μL of DTNB (10 mM) were added. The mixture was incubated under constant agitation using a shaker for 15 min at room temperature. Then, 217 μL of acetylcholine iodide (1.38 mM) was added, and the reaction mixture was left stirring for 5 min. At the end of this time, the absorbance was measured at 412 nm on the spectrophotometer. The negative control assay was performed by adding 150 μL of methanol instead of the extract.

The percentage of AChE inhibition was calculated from the following equation 1:

$$\%\mathrm{Enzyme}\;\mathrm{inhibition}=\frac{\left({\mathrm{Absorbance}}_{\mathrm{negativecontrol}}-{\mathrm{Absorbance}}_{\mathrm{sample}}\right)}{{\mathrm{Absorbance}}_{\mathrm{negativecontrol}}}\times 100$$ 1

**eq 1.** Calculation of the percentage of AChE inhibition

The tested concentrations of the extract were varied from 50 to 500 μg mL^–1^ to construct the inhibitory capacity curve, which consists of “sample concentration *versus* % enzyme inhibition,” from which the IC_50_ value, the sample concentration that inhibits 50% of the enzyme, was obtained.

### Instruments

The AChE activity and ligand fishing assay were monitored using an LC-HRMS system. The LC system used was 1290 Infinity II (Agilent Technologies, USA) consisting of a binary pump (G7120A – *High speed Pump*) and an autosampler and a column oven (G7129B – 1290 *Vial sampler*) coupled to a high-resolution mass spectrometer containing a quadrupole time-of-flight (QTOF) mass analyzer equipped with an electrospray (ESI) source. The HRMS analysis was performed by using an Impact HD QTOF mass spectrometer (Bruker Daltonics, Germany) operating in the positive ion mode. Data acquisitions were carried out using Data Analysis 4.0 software (Bruker Daltonics, Germany).

### Immobilization of AChE on Magnetic Beads and Activity Assay

Immobilization of AChE from *Electrophorus electricus* on the surface of amine-terminated magnetic beads (AChE-MBs) was adapted from the literature.^9,23^ For control-MBs, the active enzymes were inactivated after the immobilization procedure. To this end, AChE-MBs were incubated with 500 μL of methanol for 30 min under agitation. Enzyme activity for AChE-MBs and control-MBs was carried out with 5 mg of each by incubation with 200 μM acetylcholine in ammonium acetate solution (15 mM, pH 8.0) at room temperature. The reaction was interrupted using a magnetic separator; the supernatants were collected, and aliquots of 10 μL were injected into the LC-HRMS system.

The enzyme activity was monitored by the formation of choline (Ch) *m*/*z* = 104.1071 from ACh *m*/*z* = 146.1170. The activity of AChE-MBs was monitored in the presence of galantamine (1 mM, methanol) and 10 min of incubation. The AChE-MBs activity reaction was monitored using a Acquity BEH HILIC column (100 × 2.1 mm, 1.7 μm) under the isocratic elution mode with acetonitrile/ammonium acetate solution (15 mM, pH 8.0) 90:10 (v/v) at 0.4 mL min^–1^ and 35 °C. The QTOF instrument parameters were set as follows: positive ionization mode, capillary voltage, 4500 V; end plate offset, 500 V; nebulizer, 4.0 bar; dry heater temperature, 200 °C; dry gas flow, 10 L min^–1^, collision cell energy, 10 eV; transfer time, 70 μs, pre pulse time, 5 μs, and full-MS scan range, *m*/*z* 80–600.

### Sample Preparation

About 10 mg of *T. ophiraphidites* extract sample was solubilized in methanol up to a concentration of 10 mg mL^–1^ using a 20 min ultrasound bath to ensure complete solubilization. Then, an aliquot was diluted to generate a 1 mg mL^–1^ solution in methanol/ammonium acetate solution (15 mM, pH 8.0) 90:10 (v/v). Finally, the solution was sonicated for 5 min and centrifuged at 7,267 g for 5 min at a temperature of 25 °C.

### AS-MS Assay

The AS-MS assay was carried out according to previous procedures with modifications^9^ using the protocol load, wash, and extraction. In the load step, 500 μL of *T. ophiraphidites* extract sample (1 mg mL^–1^) was incubated with 5 mg of AChE-MBs for 10 min at room temperature under agitation. Then, AChE-MBs were taken to the magnetic separator, and then the supernatants (S-1) were removed, followed by washing twice with 500 μL of ammonium acetate solution (15 mM, pH 8.0). Finally, the extraction step was carried out by incubation of AChE-MBs with 500 μL of ammonium acetate solution (15 mM, pH 8.0) containing 20% methanol and acetylcholine (2 mM) for 15 min at 20 rpm and room temperature. The extraction supernatants (S-2) were collected and analyzed by LC-HRMS in the positive ionization mode. The parallel control experiment was conducted with control-MBs, and all experiments were carried out in duplicate.

For the LC-HRMS analyses, the conditions were Acquity HSS T3 reverse phase column (100 × 2.1 mm, 1.8 μm) in a gradient elution using 0.1% formic acid in water (solvent A) and ACN (solvent B) as the mobile phase, at a flow rate 0.2 mL min^–1^, at 30 °C, and 5 μL volume of injection. The total run time was 34.5 min using a linear gradient from 2 to 100% B in 25 min, followed by an isocratic step at 100% B from 25 to 27.5 min and reconditioning under the initial condition for 7 min. The ESI source operated in the positive ion mode and the QTOF parameters were set as follows: scanning experiment of total ions from *m*/*z* 50 to 1300, 4500 V capillary voltage, 500 V at the end plate, 9 L min^–1^ drying gas flow rate, nebulizer pressure (N_2_) of 2.0 bar, 180 °C temperature drying, 1 eV quadrupole ionization energy, 12 eV collision cell energy, 70 μs transfer time, and 8 μs prepulse time. LC-HRMS data were externally calibrated with sodium formate solution (1 mM) and converted to mzXML format by Bruker Compass DataAnalysis 4.2 software (Bruker Daltonics, Germany).

### AS-MS Assay Data Mining

The mzXML data obtained from the S-2 supernatants were processed in MZmine software according to algorithms and parameters described in Table 1 below.

**Table 1:** Parameters of the Algorithms Used in Data Processing by MZmine

algorithm	parameters	values
mass detection	time range	1.0 a 27.5 min
	background in MS^1^ spectra	1 × 10^3^
	mode	centroid
chromatogram builder ADAP	minimum number of scans	20
	minimum group intensity	3.5 × 10^3^
	minimum band height (intensity)	3.5 × 10^3^
	tolerated *m*/*z* difference	0.001 Da/5 ppm
automated data analysis pipeline (ADAP)	calculation of the center of *m*/*z*	median
	signal to noise ratio limit (S/N)	10
	S/N estimator	intensity window
	minimum band height	3.5 × 10^3^
	coefficient/area limit	100
	chromatographic band duration range	0.0 a 1.0 min
	chromatographic band retention time range	0.0 a 0.2 min
isotopic peaks grouper	tolerated *m*/*z* difference	0.001 Da/5 ppm
	tolerated retention time difference	0.1 min
	maximum ion charge	3
	representative isotope	lowest *m*/*z* value
join aligner	tolerated *m*/*z* difference	0.001 Da/5 ppm
	tolerated retention time	0.5 min
	weight for *m*/*z* parameter	75%
	weight for retention time parameter	25%
gap filling (peak finder)	intensity tolerance	10%
	tolerated *m*/*z*	0.001 Da/5 ppm
	tolerated retention time	0.5 min

The aligned data table was converted into .txt and imported into Excel software (Microsoft Office Professional Plus 2016) in which the affinity ratio (AR) was performed for each molecular feature (retention time, *m*/*z*) according to eq 2 described below.

$$\mathrm{Affinity}\;\mathrm{ratio}\;(\mathrm{AR})=\frac{\mathrm{Average}\;\mathrm{chomatographic}\;\mathrm{peak}\;\mathrm{area}\;\mathrm{in}\;S‐2(\mathrm{ACHE}-\mathrm{MBs})}{\mathrm{Average}\;\mathrm{chomatographic}\;\mathrm{peak}\;\mathrm{area}\;\mathrm{in}\;S‐2(\mathrm{control}-\mathrm{MBs})}$$ 2

**eq 2.** Calculation of AR of a molecular feature (retention time, *m*/*z*).

### Dereplication of AChE Ligands Present in *T. ophiraphidites* Extract

For the process of dereplication of AChE ligands, the LC-MS/MS method was used with the same chromatographic parameters previously described for the S-2 supernatants of the sponge extract. The crude sponge extract was prepared at concentrations of 1 and 2.5 mg mL^–1^ in methanol/ammonium acetate solution (15 mM, pH 8.0) 90:10 (v/v) and analyzed. The fragmentation experiments were carried out in the auto MS mode (data-dependent acquisition) for a time cycle of 3 s, with reconsideration of the ion after 0.5 min if the intensity was 2 times greater, transfer times of 40 and 80 μs, and energies for fragmentation 15–75, 20–30, 20, 30, 30–45, and 40 eV, including the ligand ions in the preference and inclusion list.

The data were converted to mzXML format using Data Analysis 4.0 software and submitted to Global Natural Products Social Molecular Network (GNPS; http://gnps.ucsd.edu). The molecular network calculations and database matching were constructed using 0.02 Da as the precursor ion mass tolerance and fragment ion mass tolerance, 0.65 as the minimum cosine score, and 3 as the minimum matched fragment ions for edge linkage. Finally, GNPS data were then imported and visualized using Cystoscope software (version 3.7.1) to find the subnetwork portions.

### Construction and Optimization of the Molecules

The 2D structures of the stereoisomers of 6-desmethyl-6-ethyl-9,10-dihydrospongosoritin A (**1A**–**D**) and the enantiomers of 3,5-dibromo-*O*-methyltyrosine (**2A**,**B**), 3-bromo-5-iodo-*O*-methyltyrosine (**3A**,**B**), and 3,5-di-iodo-*O*-methyltyrosine (**4A**,**B**) (Figure 1) were manually drawn in ChemSketch version 12.1 from the company Advanced Chemistry Development (ACD/Laboratories).^24^ All possible stereoisomers of the ligands have been considered since they were annotated from their MS data. The 3D structures were obtained in Avogadro version 1.1.1.^25^ The ionization state of the molecules was analyzed in MarvinSketch version 6.2.3 ^26^ for a physiological pH of 7.4. Finally, geometry optimization was performed using the semiempirical method AM1 ^27^ for all molecules through the extension of the MOPAC2016 software^28^ in Avogadro.^25^

**Figure 1:**
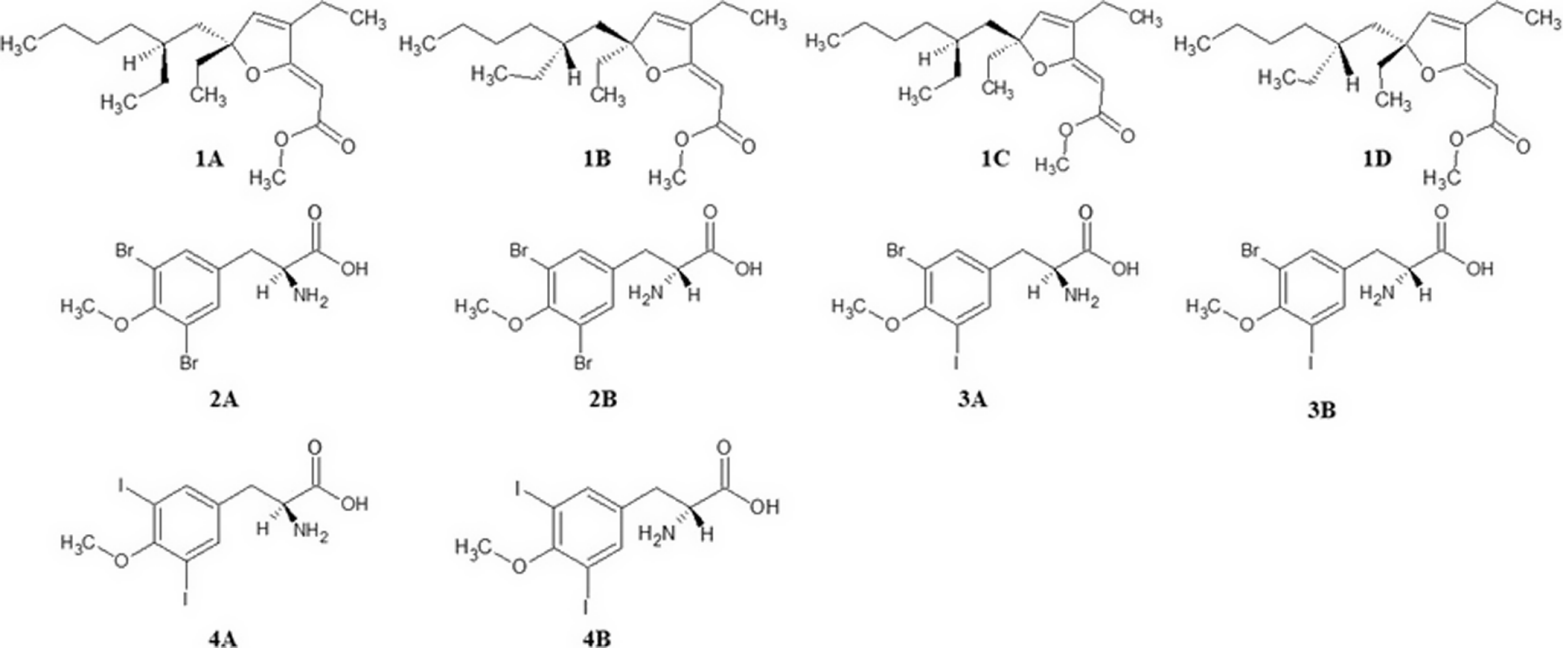
2D representation of stereoisomers **1A–** **D** of 6-desmethyl-6-ethyl-9,10-dihydrospongosoritin A and enantiomers **2A**,**B** of 3,5-dibromo-*O-*methyltyrosine, **3A,B** of 3-bromo-5-iodo-*O-*methyltyrosine, and **4A,B** of 3,5-di-iodo-*O-*methyltyrosine.

### Docking Studies and Analysis of Interactions

Molecular docking has been a useful tool to propose binding poses to biological targets and establish structure–activity relationships, among other applications.^29–32^ In this work, all molecular docking studies were performed using GOLD (Genetic Optimization for Ligand Docking) version 2024.1.0.^33^ The three-dimensional structure of human AChE complexed with donepezil^34^ was obtained from the Protein Data Bank (PDB ID = 4EY7 at 2.35 Å resolution).^34^ In the protein structure, only the A chain was kept for ligand fitting purposes, and the others were deleted. Water molecules, cofactors, and cocrystallized inhibitors contained in the PDB file were also removed.

ChemPLP scoring function^35^ was chosen to evaluate the fitting poses of the ligands based on previously validated work from our group and the literature.^36,32^ The binding site was defined as all atoms within a 15 Å radius of Phe295, which was used as a reference residue in the molecular docking study. Analysis of intermolecular interactions was performed for the ligand-AChE complexes with Pymol, v 0.99 for Windows,^37^ and Discovery Studio 2020.^38^

## Results and Discussion

### AS-MS

For prospecting AChE ligands in samples of *T. ophiraphidites* collected in the archipelago of Fernando de Noronha, Brazil, the selected sample (FN 058, Frade) was first evaluated by the Ellman’s inhibitory assay, and the methanolic extract showed an IC_50_ of 288.6 μg mL^–1^. Organic extracts of a sample of *T. ophiraphidites*, collected in the Caribbean Sea (Curaçao), were also investigated for the AChE potential by the Ellman method. The observed IC_50_ values were 31, 34, and 217 μg mL^–1^ for the acetone, butanol, and methanol extracts, respectively, evidencing that the extraction solvent directly influenced the anti-eelAChE activity.^20^

The most valued benefit of the AS-MS assay is that it focuses on the bonded target ligands, disclosing the ligands from a mixture regardless of its functional effect, and the ligands are identified by their exact mass. To meet this end, the ligands are determined by an AR or index, calculated through control assays.^4^

Herein, the AS-MS assay was carried out based on AChE-MBs. The general workflow was developed as previously described and encompasses four main steps: load, wash, ligand extraction or desorption, and LC-HRMS analysis.^8^ The assays were carried out in duplicate for the active AChE-MB and for the control. The control serves to differentiate the ligands from nonligands and, thus, discards unspecific binders. The base peak chromatograms for *T. ophiraphidites*, AChE-MB, and for the control extract are presented in Figure 2.

**Figure 2:**
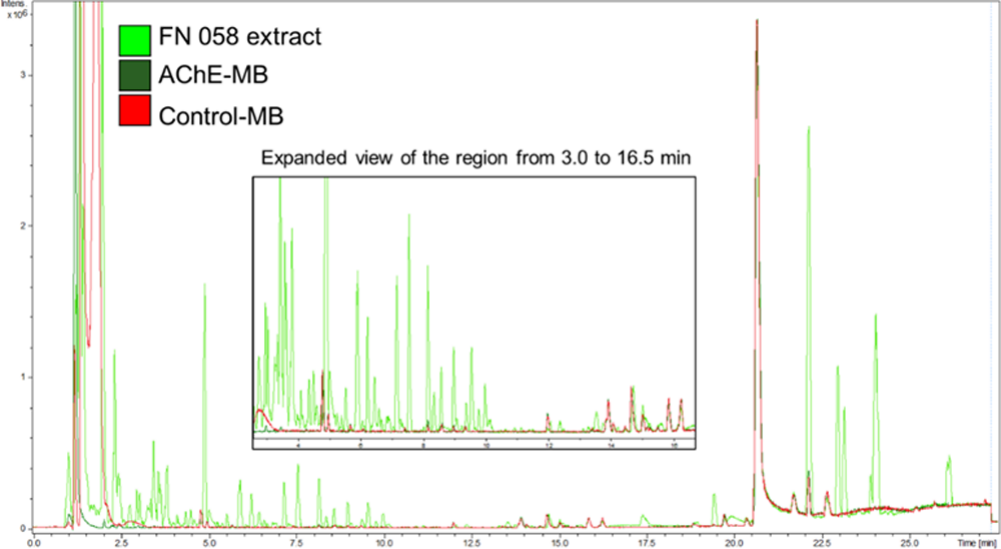
Base peak chromatograms obtained by LC-HRMS analysis for the *Topsentia ophiraphidites* extract (FN 058) and S-2 supernatants (AChE-MB and control-MB) from the ligand fishing assay.

To mine the LC-HRMS data of the desorption fraction, MZmine software was used for processing the chromatographic profiles prior to AR calculation. The threshold for AR values was established based on previous optimization assays using galantamine as a reference ligand.^8^ As the AR varied depending on the extract due to differences in the concentration of the compounds, a 20% cutoff was applied as a safety margin,^39^ considering that the calculation involves both active and inactive enzymes and that nonspecific adsorption onto particles is a known source of bias.^12^ To minimize false positives, only ligands that consistently displayed AR ≥ 1.2 across replicates and were absent or negligible in the control experiments were retained. To discard artifacts, the identified ligands were manually curated with the blank samples and the chromatographic profile of the FN 058 extract. The plot at Figure 3 shows the *m*/*z* of 14 ligands with AR varying from 1.20 to 69.55 and their corresponding retention times in the chromatogram. The ligands are of low molecular mass, below 450 Da, and with a wide range of retention times on the reverse phase elution mode, probably due to a diversity of nonpolar interactions.

**Figure 3:**
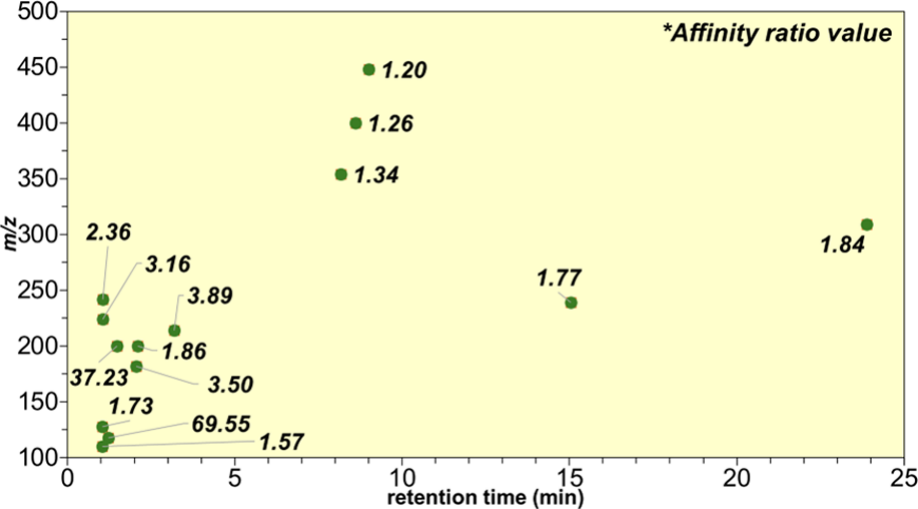
Scatter plot of AChE ligands in the *Topsentia ophiraphidites* extract.

Aiming at the structural dereplication of the identified ligands, two workflows were used: first, by the comparison of the accurate mass with the data from an in-house molecular library based on the literature of the genus and, second, by the molecular network for the annotation of molecular clusters and chemical structures by similar MS/MS spectra. Despite this procedure, only 4 out of the 14 ligands were annotated. This relatively low annotation rate highlights the unexplored chemical diversity of this sponge. Although automated tools greatly enhance the efficiency of dereplication, their limitations remain evident, particularly with stereoisomers or compounds absent from existing databases, and even a complete extract dereplication does not guarantee that the compounds identified in AS-MS are the same as those annotated during dereplication.^40^ Thus, for the four annotated ligands, the retention times, the precursor ions (experimental and theoretical), and the MS/MS information are shown in Table 2 according to their decreasing ARs.

**Table 2:** Chemical Profile of *Topsentia ophiraphidites* Extracts: Information on Dereplicated Substances

#	name	RT (min)	experimental/theoretical (*m*/*z*)	ion form	molecular formula	error (ppm)	ions in MS^2^ spectra	AR
**1**	6-desmethyl-6-ethyl-9,10-dihydrospongosoritin A	23.77	309.2420/309.2424	[M + H]^+^	C_19_H_32_O_3_	–1.3	309.2416, 295.2258, 277.2157, 259.2049, 249.2203, 231.2100, 217.1589, 203.1430, 179.1059, 163.1472, 151.1107, 149.0226, 137.0955, 121.1004, 119.0851, 109.1005	1.84 ± 0.19
**2**	3,5-dibromo-*O*-methyltyrosine	8.22	353.9165/353.9158	[M + H + 2]^+^	C_10_H_11_Br_2_NO_3_	2.0	355.9137, 353.9161, 351.9179, 338.8866, 336.8895, 334.8913, 30.90986, 307.9110, 305.9130, 228.9921, 226.9942, 213.9688, 211.9708, 187.9653, 185.9675, 147.0677, 133.0518, 132.0445, 105.0572	1.34 ± 0.04
**3**	3-bromo-5-iodo-*O*-methyltyrosine	8.67	399.9033/399.9040	[M + H]^+^	C_10_H_11_BrINO_3_	–1.8	401.9016, 399.9029, 384.8736, 382.8778, 355.8961, 353.8985, 274.9792, 257.9706, 255.9721, 228.9916, 226.9930, 213.9684, 211.9701, 148.0748	1.26 ± 0.00
**4**	3,5-diiodo-*O*-methyltyrosine	9.04	447.8913/447.8901	[M + H]^+^	C_10_H_11_I_2_NO_3_	2.6	447.8909, 430.8639, 401.8846, 303.9584	1.20 ± 0.05

Among the four ligands identified through the AS-MS experiment, one was a polyketide featuring a dihydrofuran ring, identified as 6-desmethyl-6-ethyl-9,10-dihydrospongosoritin A (**1**), and the other three were halogenated aromatic amino acids, 3,5-dibromo-*O*-methyltyrosine (**2**), 3-bromo-5-iodo-*O*-methyltyrosine (**3**), and 3,5-diiodo-*O*-methyltyrosine (**4**). Despite the use of GNPS, none of these compounds was directly matched through the spectral libraries available on the platform.

Nevertheless, dereplication of the crude extract (data not shown) provided valuable insights through molecular networking, which revealed clusters that supported structural proposals for these four ligands. Ligand **1** was found within a cluster that included spongosoritin A and other derivatives previously reported in sponges of the Plakinidae family.^41^ The structural similarity allowed its identification by comparing the MS^2^ spectra obtained from the *T. ophiraphidites* extract with those described in the literature as losses of CH_3_OH, CO, H_2_, CH_4_, and remote hydrogen rearrangement. Spongosoritins are compounds of significant biological interest due to their cytotoxic activity.^42^ Despite the presence of spongosoritin A and other polyketides in the molecular cluster, only *m*/*z* 309.2415 was selectively fished. We did not find any reports of this molecule as an AChE ligand.

Regarding the halogenated aromatic amino acids, the molecular networking of the extract revealed two clusters corresponding to this class of compounds. These clusters indicated the presence of 3-iodo-tyrosine and 3,5-diiodo-tyrosine, both previously reported in the marine sponges *Aplysina cavernicola* ^43^ and *Ianthella basta*.^44^ In this way, ligands **2** and **4** were proposed based on their structural similarity, molecular formulas, fragmentation patterns, and searches in the Dictionary of Marine Natural Products. Ligand **3**, on the other hand, was proposed as a novel compound, structurally related to nonmethylated tyrosine analogues previously isolated from the same sponges *Aplysina cavernicola* ^43^ and *Ianthella basta*.^44^

To date, there are no reports of halogenated amino acids as an AChE ligand, such as bromo- tyrosine and iodo-tyrosine, but it is possible to find a number of citations to bromo-tyrosine alkaloid derivatives as AChE inhibitors.^45,46^ Other halogenated amino acids are present on the network cluster of the *T. ophiraphidites* extract, and six others were dereplicated, but only these three aforementioned were present on the desorbate of the AS-MS assay showing the selectivity of the used screening.

#### Docking Studies of the Dereplicated Ligands

The binding site of AChE has been thoroughly investigated by either experimental or theoretical structure-based methods. It is comprised of a deep and narrow gorge with a few subsites, including the catalytic anionic site (CAS), the oxyanion hole (OH), and the peripheral anionic site (PAS).^47^ Among them, the most important is the CAS at the bottom of the binding site and the PAS at the entrance of the gorge. PAS is responsible for the initial recognition of cationic substrates and can also allosterically modulate functionalized surface activity. In addition, PAS is also involved in noncholinergic functions, such as amyloidosis, neurite outgrowth, and cell adhesion.^48^ Substrates, first interacting with PAS, can be led all the way through the gorge toward the CAS, at the bottom, aided by amino acid residues from other subsites. It is worth noting that the amino acid residues that make up the PAS of AChE are Tyr72, Asp74, Tyr124, Trp286, and Tyr341. In addition, the CAS is surrounded by the amino acids Trp86, Tyr133, Glu202, and Phe338 and the catalytic triad represented by Ser203, Glu334, and His447.^47^

As stated in the Experimental Section, docking parameters have been established previously in our research group, and the top redocking pose of donepezil and RMSD values are reproduced in Table S1 and Figure S1 of the supporting material. In this work, docking scores (Figure S2, Table S2) aligned with AR values observed for the dereplicated ligands (Table 2), being higher for **1A–** **D** compared to the other studied stereoisomers. In addition, our study shows that all stereoisomers bind deeply into the active site gorge (Figures 4, 5, and S3–S10).

**Figure 4:**
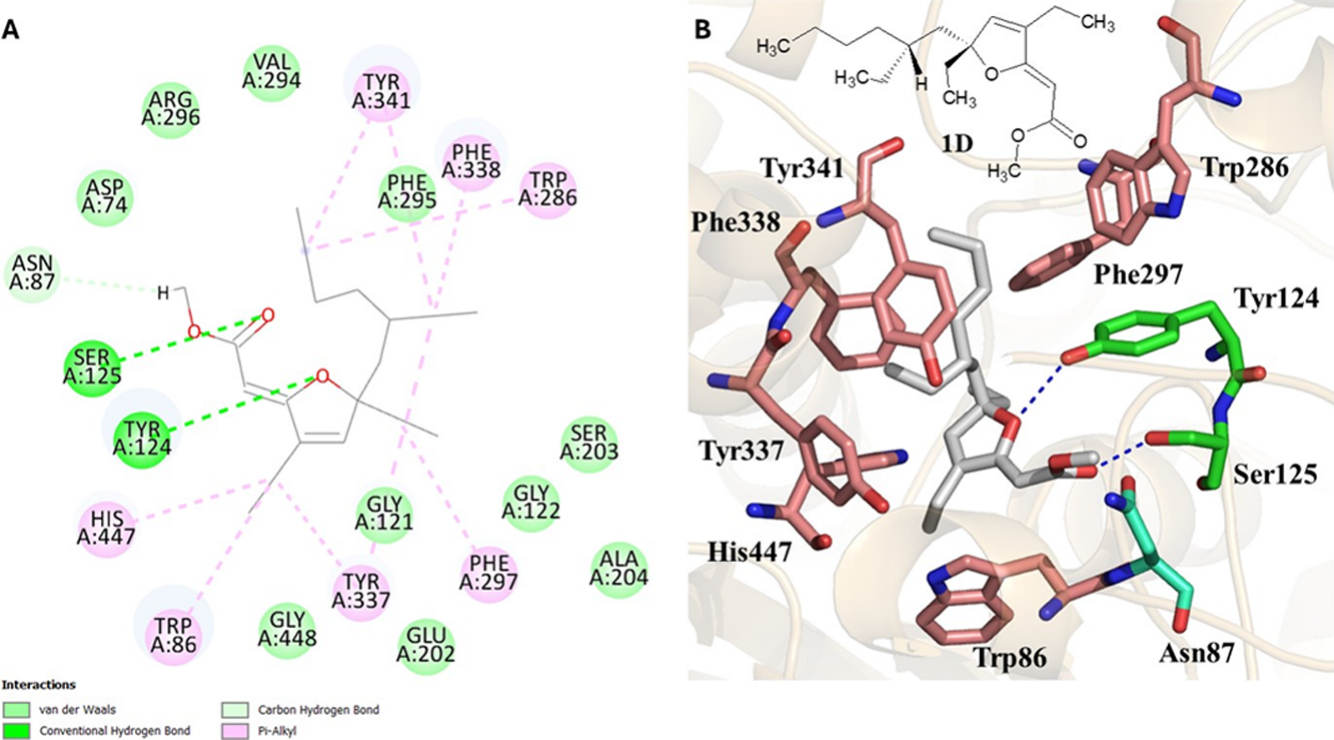
2D and 3D representations of stereoisomer **1D** and its interactions at the AChE active site. In A, representation of the 2D diagram showing the types of interactions with the respective residues. In part B, representation of **1D** in 2D and 3D with gray carbon atom sticks and the respective interacting residues.

**Figure 5:**
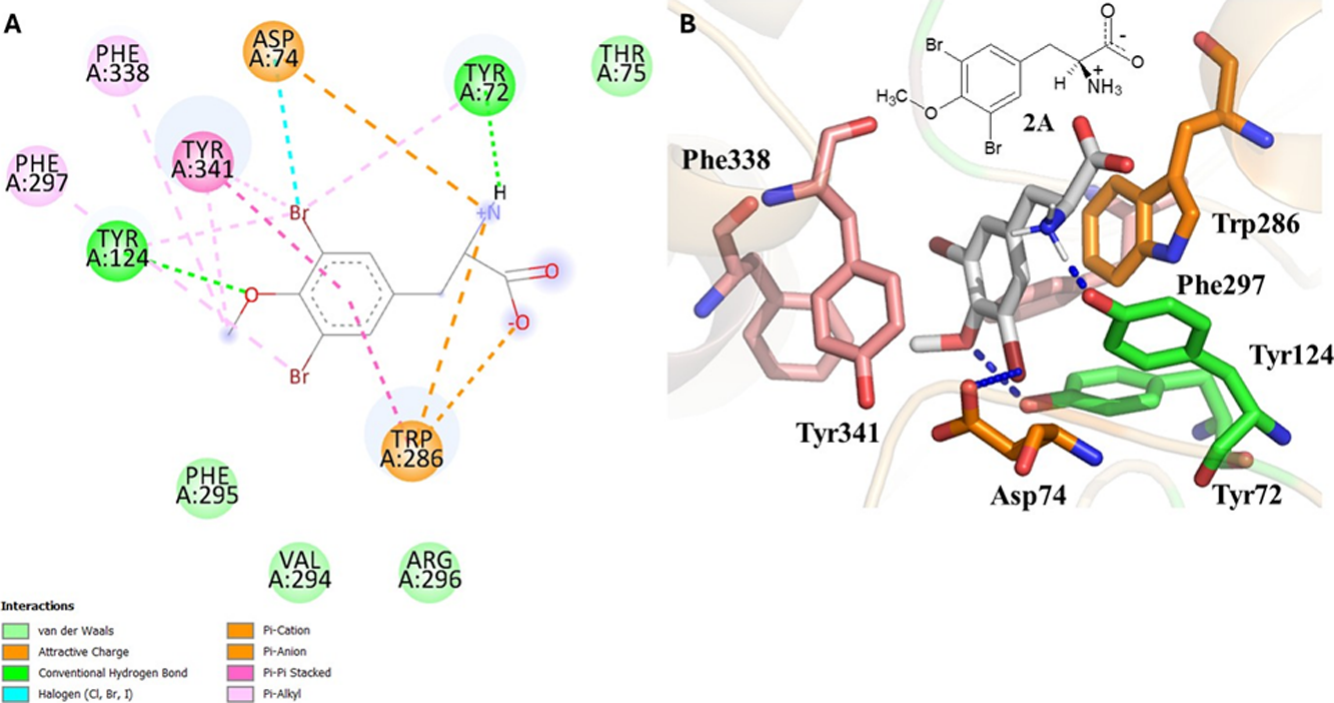
2D and 3D representations of enantiomer **2A** and its interactions at the AChE active site. In A, representation of the 2D diagram showing the types of interactions with the respective residues. In B, representation of **2A** in 2D and 3D with gray carbon atom sticks and the respective interacting residues.

The four stereoisomers of 6-desmethyl-6-ethyl-9,10-dihydrospongosoritin A (**1A–** **D**) performed at least one type of interaction with the residues of PAS, CAS, and the catalytic triad (Figures 4, S3–S5). However, stereoisomer **1D** (*R*, *R*) stood out the most, having presented the highest score of 86 (Figure S2) in the series of stereoisomers, which aligns with its highest AR value among the dereplicated compounds (Table 2). Interactions with PAS residues were observed between the oxygen atom of the furan ring of **1D** and Tyr124 by conventional hydrogen bonding and Trp286 and Tyr341 by π-alkyl nonpolar interactions with the hydrocarbon side chain. Trp86 and Phe338 (CAS) also showed π-alkyl nonpolar interactions involving the hydrocarbon skeleton of the ligand. This same type of interaction was also observed with His447, which is part of the catalytic triad, and with other residues (Tyr337 and Phe297), which are not part of the subsites already described. Another type of interaction was observed with Ser125 by conventional hydrogen bonding with the oxygen atom of the carbonyl of the ester group and with Asn87, which interacted by carbon–hydrogen bonding with one of the methyl hydrogens of the ester group. Finally, unspecific van der Waals (dispersion) interactions were noted with residues Asp74 (PAS), Phe295 (CAS), and Ser203 (catalytic triad) and with other residues, such as Val294, Arg296, Gly448, Gly121, Glu202, Gly122, and Ala204.

The enantiomers of 3,5-dibromo-*O*-methyltyrosine (**2A,B**) also performed at least one type of interaction with PAS, CAS, and catalytic triad residues (Figures 5 and S6). Since both enantiomers presented equivalent score values of 62 and 63 (Figure S2) and because enantiomer **2A** would most likely be formed due to the *L*-amino acid side chain, its putative interactions are described. For example, PAS residue Asp74 interacted by halogen binding and performed an electrostatic interaction of the attractive charge type involving one of the oxygen atoms of the carboxylate in the side chain of this residue and the positively charged amino group of the ligand Tyr72, which interacted by conventional hydrogen bonding and performed π-alkyl nonpolar interaction and Trp286, which performed two electrostatic interactions, one of the π-cation type and the other of the π-anion type. In addition, it performed two nonpolar interactions of the π-stacking type; Tyr124 interacted by conventional hydrogen bonding and π-alkyl and Tyr341 by nonpolar interactions of π and π-alkyl stacking type. It was also possible to observe interactions with CAS residues, for example with Phe338, that performed nonpolar interactions of the π-alkyl type and π-stacking, and with residue Phe297, which is not part of the main subsite. In addition to these interactions, van der Waals (dispersion) unspecific contacts have also been observed with Phe295 (CAS) and with Thr75, Val294, and Arg296.

Also, we observed interactions with the π system of residue Phe297, which is not part of the aforementioned AChE subsites and involved the bromine halogen atom. Finally, it was observed through molecular docking that **2A** and **2B** were the only ligands that did not interact with Tyr337, a residue considered critical for the inhibition of the human enzyme, although it also does not belong to any of the subsites.^49^

For the *S* enantiomer of 3-bromo-5-iodo-*O*-methyltyrosine (**3A**), we observed hydrogen bonding interactions involving PAS residues Tyr72 and the carboxylate and Tyr124 and the oxygen atom of the 4-methoxyphenyl substituent (Figure S7). In addition, Phe295 performed a halogen bond with the 3-iodo substituent of the phenyl ring, as did PAS residue Asp74 with the bromine. Further interactions were of the π-π type, involving PAS residues Trp286, Tyr341, and the phenyl ring, and π-alkyl interactions involving Tyr72, Tyr124, Tyr341 and the 5-Br substituent and Phe338 (CAS) and the 3-I substituent of the phenyl ring. Surprisingly, Trp286 (PAS) performed a π-cation interaction with the amino group and a π-anion interaction with the carboxylate of the side chain of molecule **3A**. In addition, for the *R* enantiomer of 3-bromo-5-iodo-*O*-methyltyrosine (**3B**) (Figure S8), very similar interactions were observed related to its enantiomer **3A** except for the important π-cation and π-anion interactions between the amino and carboxylate groups of the side chain and PAS residue Phe286. This behavior is in agreement with the score values of 60 and 61 for **3A** and **3B**, respectively (Figure S2).

Finally, the *S* enantiomer of 3,5-di-iodo-*O*-methyltyrosine (**4A**), performs interactions similar to those of the *S* enantiomer of 3-bromo-5-iodo-*O*-methyltyrosine (**3A**), including the important π-cation and π-anion interactions involving the amino and carboxylate groups of the side chain and PAS residue Phe286 (Figure S9). In addition, the *R* enantiomer of 3,5-di-iodo-*O*-methyltyrosine (**4B**) performs similar interactions, except for the π-cation and π-anion interactions involving the amino and carboxylate groups of the side chain of PAS residue Phe286 (Figure S10). It is worth noting that as observed for other stereoisomers, **4A,B** showed similar docking scores of 59 and 60, respectively (Figure S2).

In short, all molecules under study showed interactions involving π systems, such as π-alkyl, with PAS and CAS residues. Interactions of this type are reported to be relevant to the functional and structural role of proteins.^50^ These interactions, although having a smaller energy contribution compared to hydrogen bonds, for example, are generally established in greater numbers, allowing molecules to explore nonpolar contacts with PAS residues and definitely contributing to the binding free energy. This condition decreases the probability of the substrate reaching the active site of the protein since the hydrolysis process occurs at the bottom of the cavity. Furthermore, when interactions occur with PAS aromatic residues, the primary binding of acetylcholine is prevented, which is essential for it to reach the active site.

Donepezil, for example, is a highly potent synthetic drug, classified as a noncompetitive reversible inhibitor, which interacts with PAS and CAS through aromatic interactions, although without any direct interactions with the catalytic triad. Several researchers have already reported that interactions with Trp86, which is located in the CAS, are essential for the inhibition of AChE by donepezil since this residue is involved in the recognition of the substrate ACh. Phe338, on the other hand, interacts with the nitrogen in the piperidine ring of donepezil, thus serving as a “bridge” between PAS and CAS of the active site. In addition, donepezil interacts with Trp286, which is part of the PAS, where the initial binding with the substrate occurs and which is essential for it to reach the active site.^51^ All of these residues are present in interactions with molecules **1D** and **2B**, which were the ones selected for 3D depiction at the active site of AChE.

It is also worth noting that all of the molecules were able to establish interactions with Trp286, which is considered an important amino acid residue in PAS, when it comes to the molecular recognition process of the ligand, as already pointed out. Some drug candidates based on the structure of donepezil, for example, which did not show this type of interaction with Trp286, showed low inhibitory capacity.^52,53^

Furthermore, it is noteworthy that all molecules interacted by at least one of these π-alkyl interactions or hydrogen bonds with residues outside the AChE active site. Finally, all of them showed nonpolar interactions with residues that do not belong to PAS, CAS, catalytic triad, and the oxyanionic cavity, which may further contribute to binding to the enzyme.

## Conclusions

In this work, an AS-MS assay with AChE-MB has been used to search for ligands in samples of the sponge *Topsentia ophiraphidites* collected in the archipelago of Fernando de Noronha, Brazil. Ligand dereplication disclosed 6-desmethyl-6-ethyl-9,10-dihydrospongosoritin A, 3,5-dibromo-O-methyltyrosine, 3-bromo-5-iodo-*O*-methyltyrosine, and 3,5-di-iodo-*O*-methyltyrosine as AChE ligands, which showed ARs of 1.84, 1.34, 1.26, and 1.20, respectively, in an AS-MS assay. In addition, in the docking studies with AChE, the (*R*,*R*) stereoisomer of 6-desmethyl-6-ethyl-9,10-dihydrospongosoritin A, **1D**, stood out for having the highest score value, which can be related to interactions with important residues in the active site of the enzyme, such as His447, which is part of the catalytic triad. Also, more energy-contributing types of interactions may be involved in the theoretically better affinity for the active site of AChE shown by **1D** and deserve further experimental investigation.

These findings provide the first molecular evidence of AChE-binding metabolites from *T. ophiraphidites*, underscoring the value of coupling AS-MS with molecular docking to explore marine natural products. Beyond enriching the chemical knowledge base of an under-investigated sponge, this integrated approach may accelerate the discovery of novel AChE agents, with potential relevance for neurodegenerative disease research. Future studies should prioritize the isolation of these ligands to establish their configuration and whether they are orthosteric ligands. These results can contribute as a framework for medicinal chemistry to design and optimize new anticholinergic scaffolds, in the end contributing to both innovation in drug discovery and marine natural products research.

## Supplementary Material



## Notes

Redocking data, interaction types of amino acids of AChE with all compounds, and 2D diagrams and top 3D poses of compounds **1A–** **C**, **2B**, **3A**,**B**, and **4A**,**B** with AChE (PDF)
