# Supplementary material for: Affinity Selection-Mass Spectrometry for the Identification of Ligands of Acetylcholinesterase from Topsentia ophiraphidites and Docking Studies for the Dereplicated Ligands
Source: ACS Omega. 2025 Oct 14;10(42):50275–84. doi: 10.1021/acsomega.5c06784 (PMC12573006; doi:10.1021/acsomega.5c06784)
Supplement: Supplementary file 1 [file ao5c06784_si_001.pdf]

Affinity selection-mass spectrometry for the identification of ligands of Acetylcholinesterase from *Topsentia ophiraphidites* and docking studies for the dereplicated ligands

Larissa Ramos Guimarães da Silva[a], Christyne Barros de Sá[b], Bruno Sergio do Amaral[c], Nelilma Correia Romeiro[b**], Quezia Bezerra Cass[c], Alessandra Leda Valverde[a**]

[a*]*Laboratório de Produtos Naturais (LaProMar), Instituto de Química, Universidade Federal Fluminense, Niterói, RJ, 24020-005, Brazil*

[b**]*Laboratório Integrado de Computação Científica – LICC, Centro Multidisciplinar da UFRJ Macaé, Universidade Federal do Rio de Janeiro, RJ, Brazil*

[c]*Instituto Federal de Educação, Ciência e Tecnologia de São Paulo – Campus Pirituba, São Paulo, SP, Brazil*

[c]*Separare, Departamento de Química, Universidade Federal de São Carlos, São Carlos, SP, Brazil*

*alessandravalverde@id.uff.br

ORCID ID https://orcid.org/0000-0002-1250-1051

** nelilmaromeiro@gmail.com

ORCID ID https://orcid.org/0000-0003-2562-8331

Table S1. RMSD values obtained with redocking of donepezil into its crystallographic structure (4EY7) considering different binding site radius definitions around Phe295.

REDOCKING – ChemPLP			
Radius (Å)	10	15	20
RMSD (Å)	0.7	0.5	0.5

Source: reference (36).

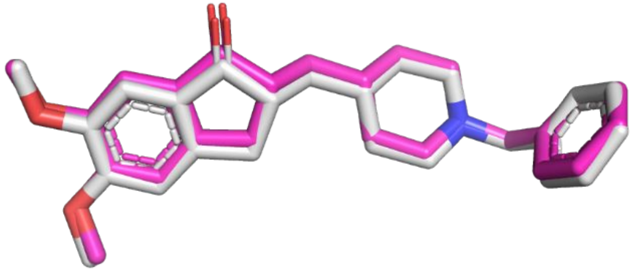

Figure S1. Superimposition between the top pose of donepezil obtained by redocking considering 15 Å binding site radius definition around Phe295 and ChemPLP scoring function (pink carbon atoms) and the crystallographic pose (gray carbon atoms). Adapted from reference (36).

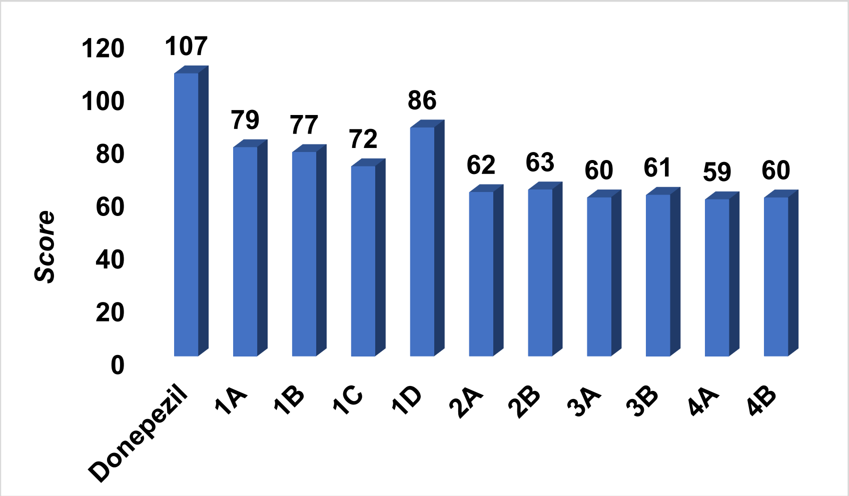

Figure S2. Score values observed for stereoisomers **1A-D** of 6-desmethyl-6-ethyl-9,10-dihydrospongosoritin A, enantiomers **2A-B** of 3,5-dibromo-*O*-methyltyrosine, **3A-B** of 3-bromo-5-iodo-*O*-methyltyrosine, **4A-B** of 3,5-di-iodo-*O*-methyltyrosine and donepezil obtained with the ChemPLP function in GOLD docking software.

Table S2. Interaction types and amino acid residues from the active site of AChE observed in the docking studies for the stereoisomers of the dereplicated ligands.

	**Selected Interaction Types**							
**Molecule**	**Hydrogen Bond**	**Halogen**	**π- π stacked**	**π -alkyl**	**π -cation**	**π -anion**	**π -sigma**	**Attractive charge**
**1A**	**Tyr124**<br>Ser125	-	-	**Trp86, Tyr124,** Phe297, **Tyr341**	-	-		
**1B**	-	-	-	**Trp86, Tyr124, Trp286,** Tyr337, **Phe338, Tyr341**	-	-	**Trp86**<br>**Tyr341**	
**1C**	**Tyr124**<br>Ser125<br>**Asp74** (carbon HB)	-	-	**Trp286, Phe338, Tyr341, His447**	-	-	-	
**1D**	**Tyr124**<br>Ser125<br>Asn87(carbon HB)	-	-	**Trp86, Trp286**, Phe297, Tyr337, **Phe338, Tyr341, His447**	-	-	-	
**2A**	**Tyr72**, **Tyr124**	**Asp74**	**Trp286**<br>**Tyr341**	**Tyr72, Tyr124**, Phe297, **Phe338, Tyr341**	**Trp286**	**Trp286**	-	**Asp74**
**2B**	**Tyr124**<br>Ser293 (HB & Carbon HB)<br>**Phe295**<br>**Tyr341**	**Phe295**	**Trp286**	**Tyr72, Tyr124, Trp286,** Phe297, **Phe338, Tyr341**	**-**	-	-	-
**3A**	**Tyr72, Tyr124, Phe295**	**Asp74**<br>**Phe295**	**Trp286**<br>**Tyr341**	**Tyr72, Tyr124**, Phe297, **Phe338, Tyr341**	**Trp286**	**Trp286**		

**3B**	**Tyr72, Tyr124, Phe295**	**Asp74 Phe295**	**Trp286**	**Tyr72, Tyr124, Trp286**, Phe297, **Phe338, Tyr341**				
**4A**	**Tyr72, Tyr124, Phe295**	**Asp74 Phe295**	**Trp286 Tyr341**	**Tyr72, Tyr124**, Phe297, **Phe338, Tyr341**	**Trp286**	**Trp286**		
**4B**	**Tyr72, Tyr124, Phe295**	**Asp74 Phe295**	**Trp286**	**Tyr72, Tyr124, Trp286**, Phe297, **Phe338, Tyr341**				

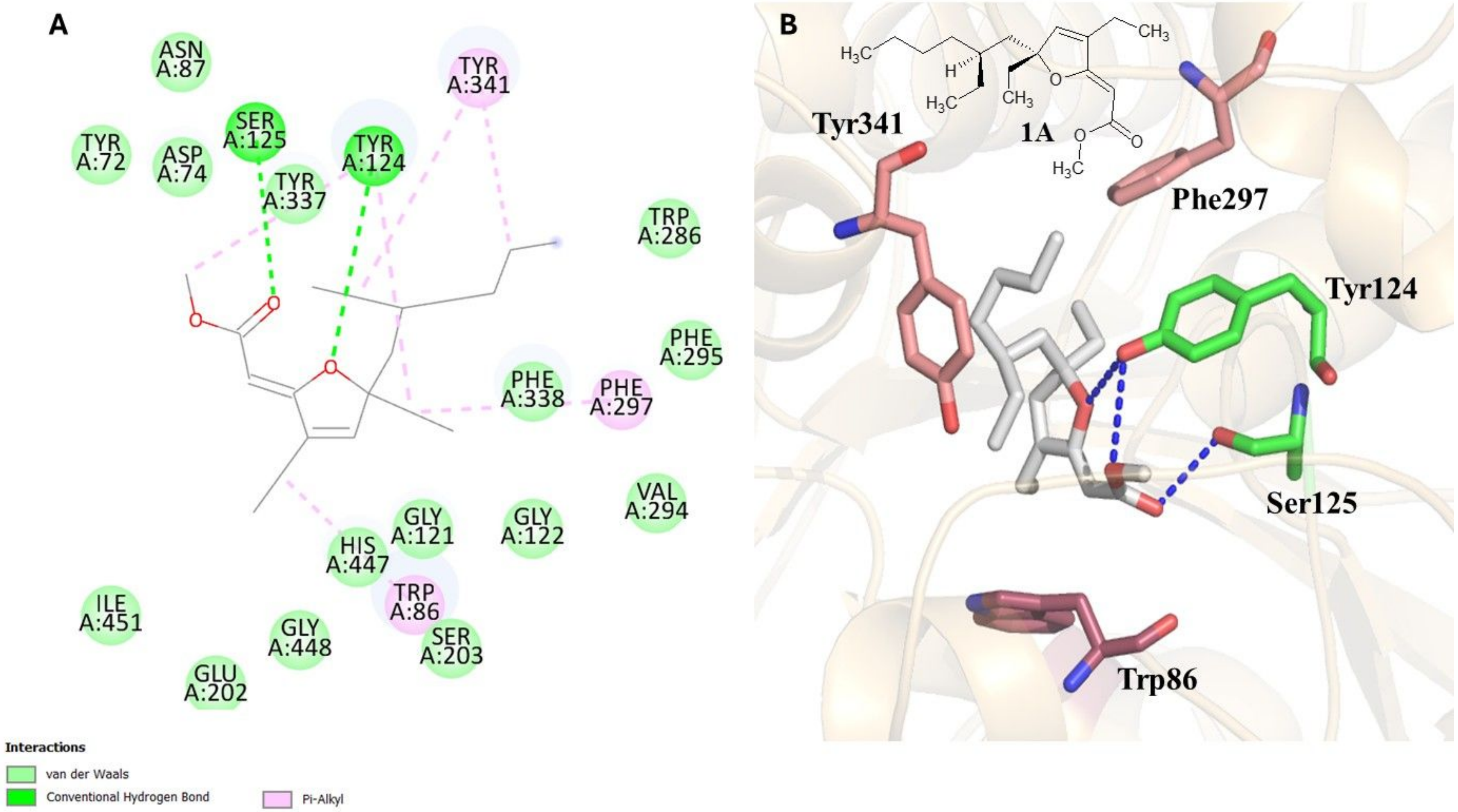

Figure S3. 2D interactions of molecule **1A** at the active site of AChE (**A**) and selected amino acid interactions in 3D (**B**).

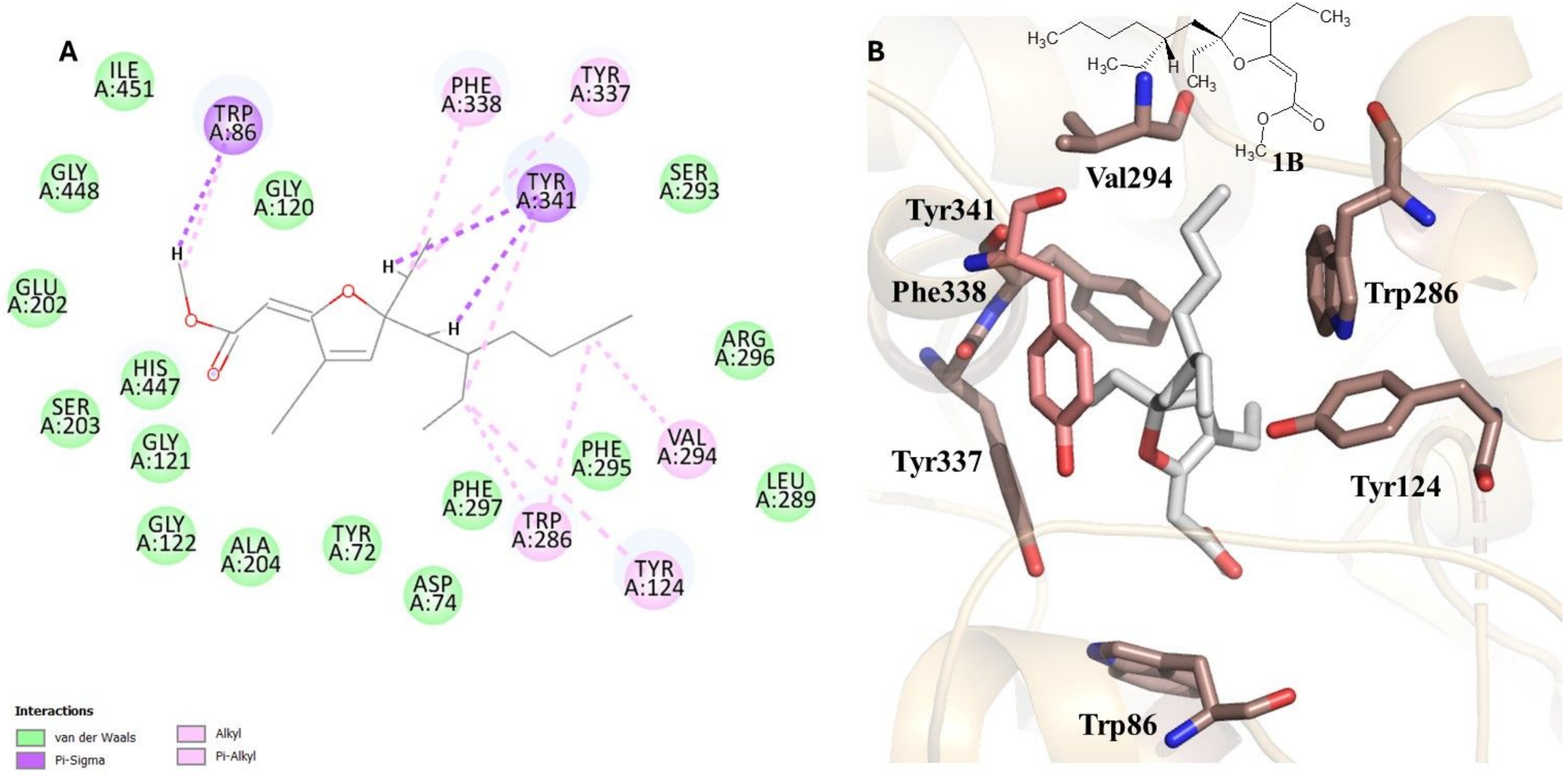

Figure S4. 2D interactions of molecule **1B** at the active site of AChE (**A**) and selected amino acid interactions in 3D (**B**).

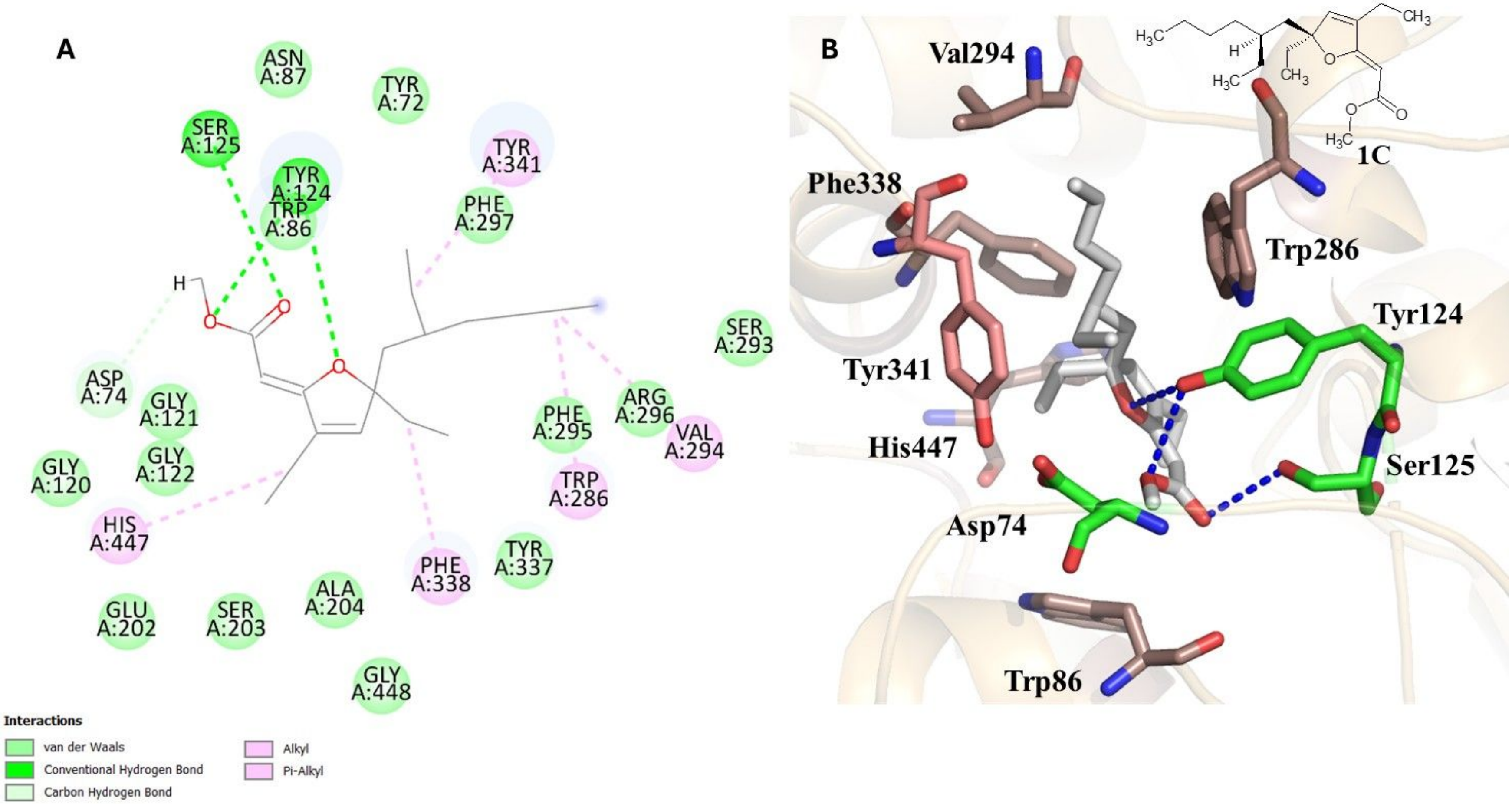

Figure S5. 2D interactions of molecule **1C** at the active site of AChE (**A**) and selected amino acid interactions in 3D (**B**).

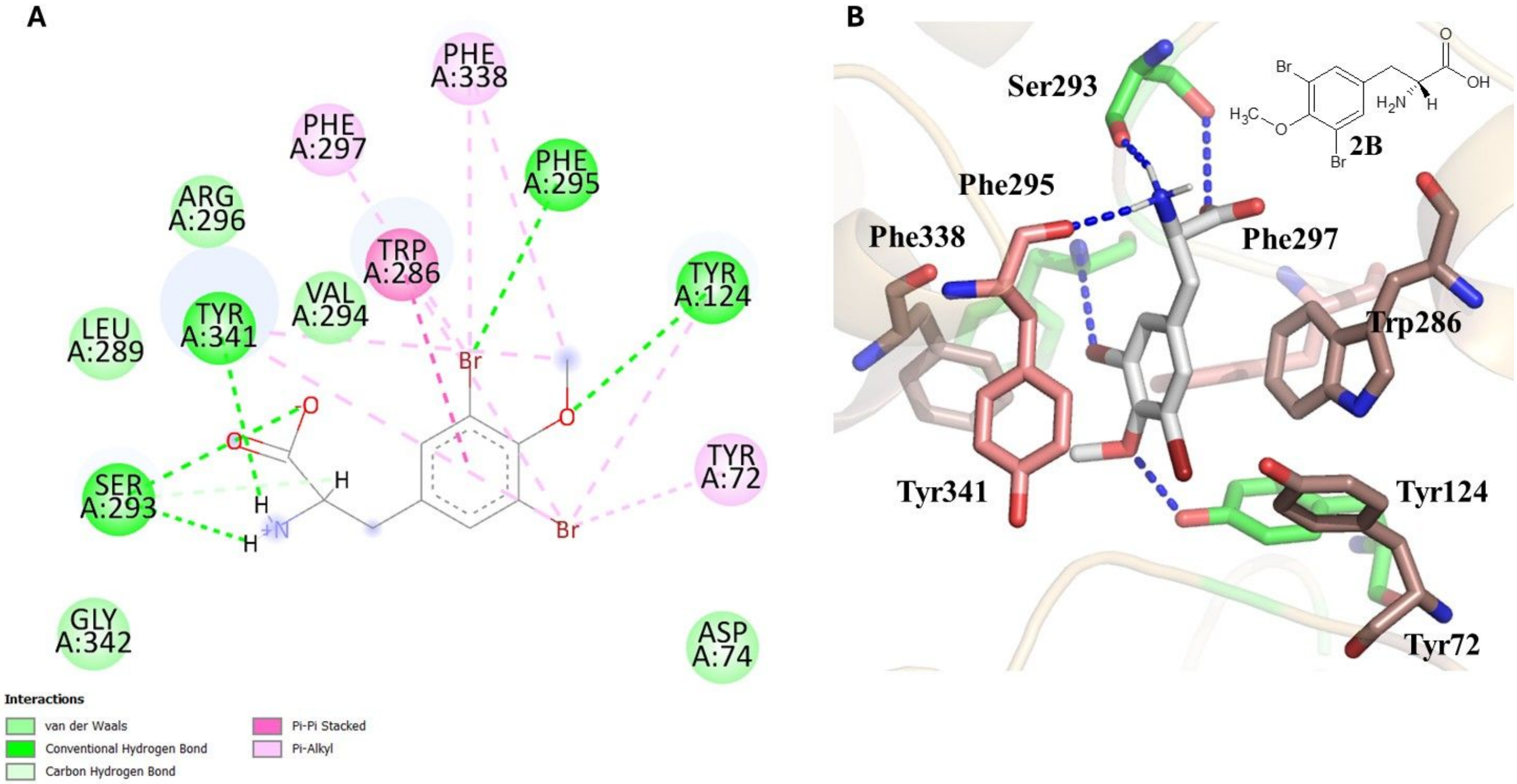

Figure S6. 2D interactions of molecule **2B** at the active site of AChE (**A**) and selected amino acid interactions in 3D (**B**).

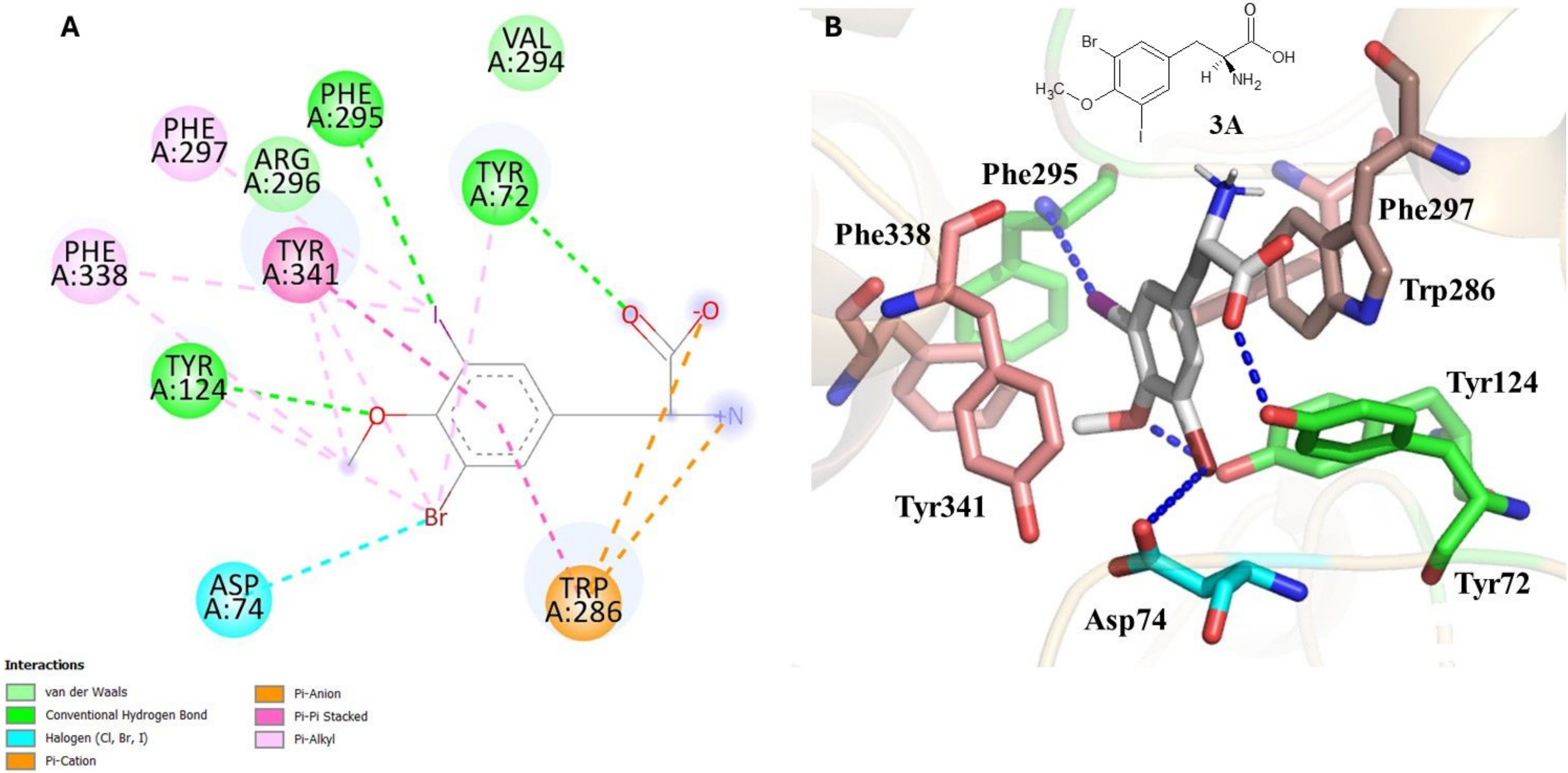

Figure S7. 2D interactions of molecule **3A** at the active site of AChE (**A**) and selected amino acid interactions in 3D (**B**).

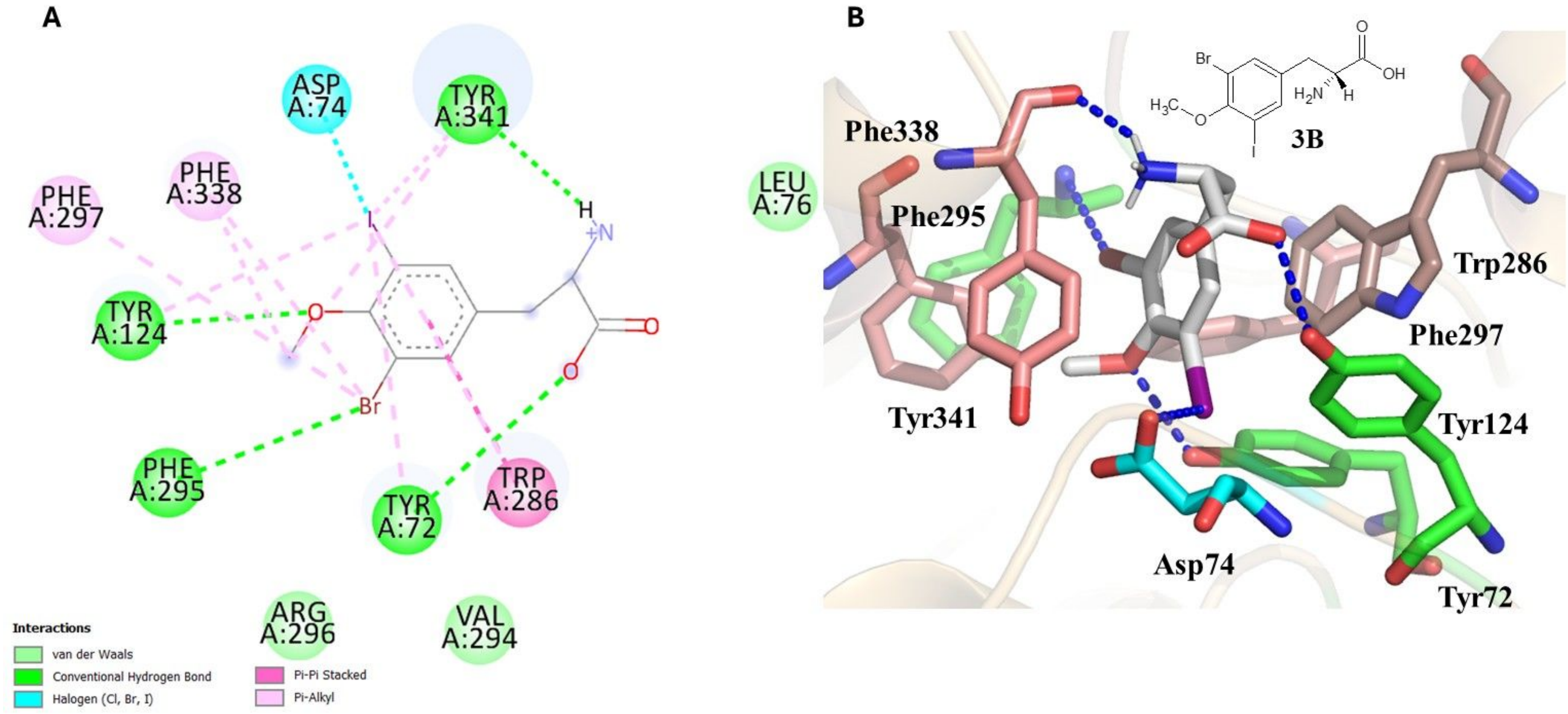

Figure S8. 2D interactions of molecule **3B** at the active site of AChE (**A**) and selected amino acid interactions in 3D (**B**).

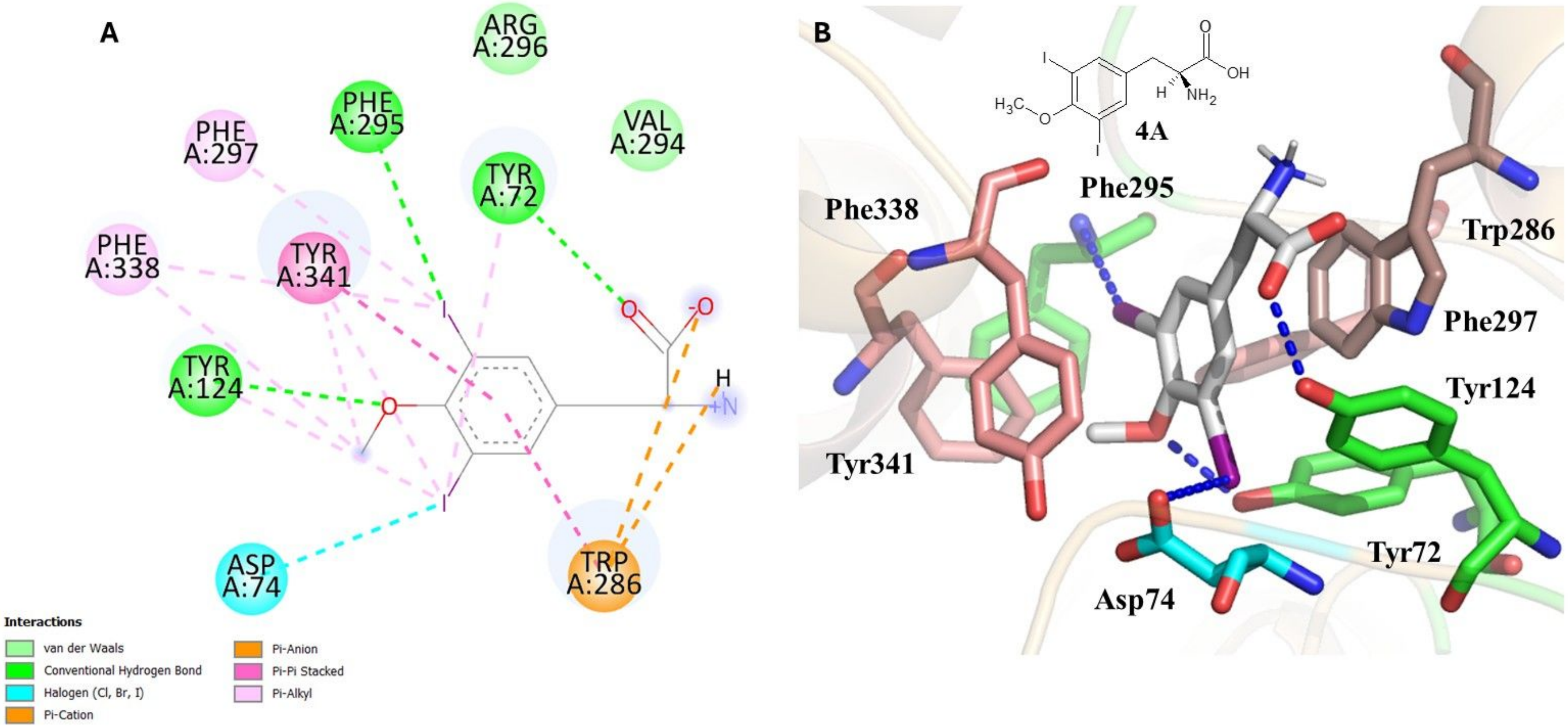

Figure S9. 2D interactions of molecule **4A** at the active site of AChE (**A**) and selected amino acid interactions in 3D (**B**).

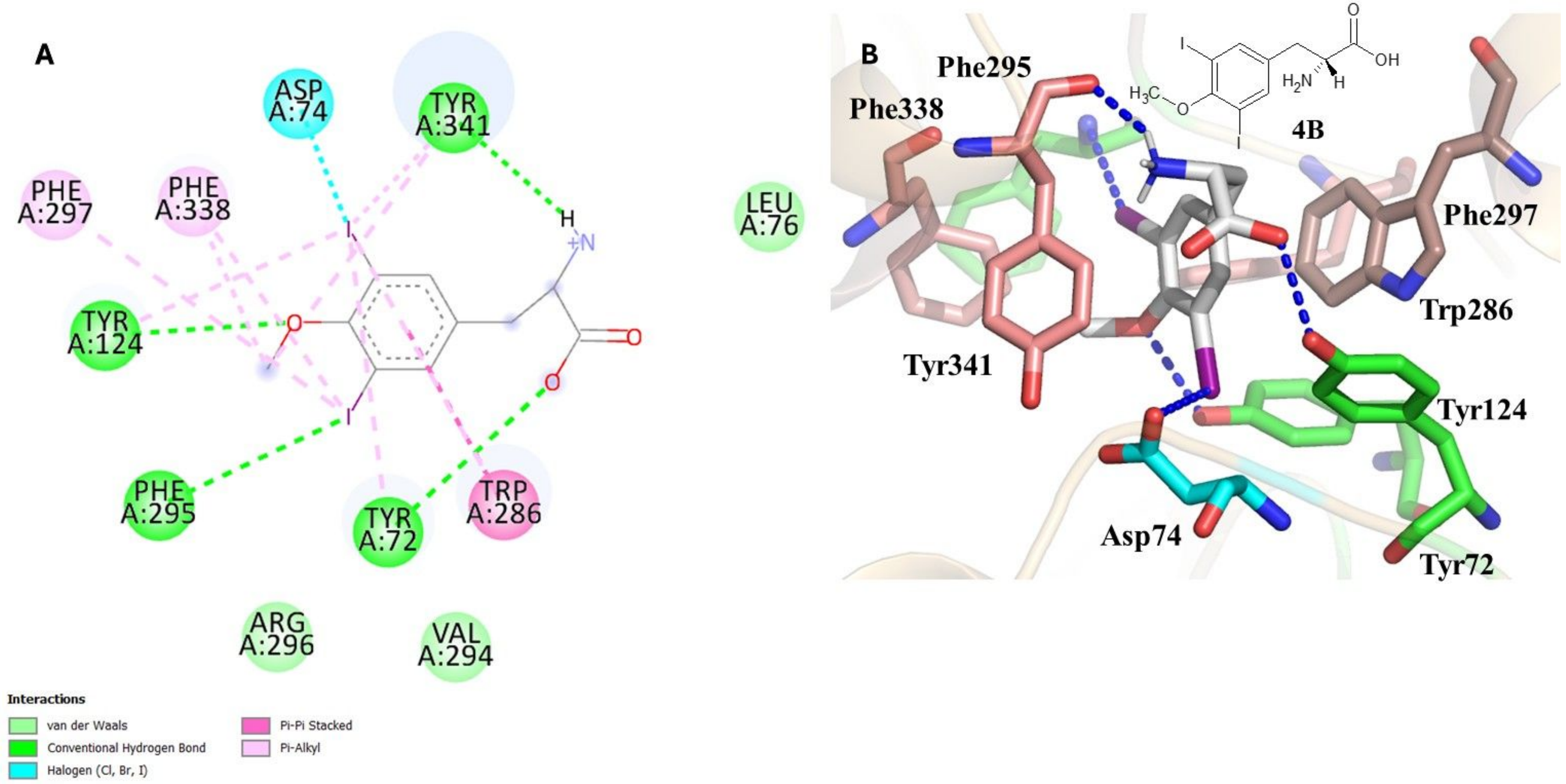

Figure S10. 2D interactions of molecule **4B** at the active site of AChE (**A**) and selected amino acid interactions in 3D (**B**).
